# On-line hemodiafiltration modulates atherosclerosis signaling in peripheral lymphomonocytes of hemodialysis patients

**DOI:** 10.1007/s40620-020-00958-z

**Published:** 2021-03-24

**Authors:** Simona Simone, Annarita Chieti, Paola Pontrelli, Federica Rascio, Giuseppe Castellano, Giovanni Stallone, Barbara Infante, Loreto Gesualdo, Giuseppe Grandaliano, Giovanni Pertosa

**Affiliations:** 1 Nephrology, Dialysis and Transplantation Unit, Department of Emergency and Organ Transplantation, University of Bari “A. Moro”, Piazza G. Cesare 11, 70122 Bari, Italy; 2 Nephrology, Dialysis and Transplantation Unit, Department of Medical and Surgical Sciences, University of Foggia, Foggia, Italy; 3 Nephrology Unit, Department of Medical and Surgical Sciences, Fondazione Policlinico Universitario “A. Gemelli” IRCCS, Rome, Italy; 4 Department of Translational Medicine and Surgery, Università Cattolica del Sacro Cuore, Rome, Italy

**Keywords:** Gene expression, Lymphomonocytes, Hemodialysis, Atherosclerosis, Cardiovascular disease

## Abstract

**Background:**

Hemodialysis patients present a dramatic increase in cardiovascular morbidity/mortality. Circulating immune cells, activated by both uremic milieu and dialysis, play a key role in the pathogenesis of dialysis-related vascular disease. The aim of our study was to identify, through a high-throughput approach, differences in gene expression profiles in the peripheral blood mononuclear cells (PBMCs) of patients treated with on-line hemodiafiltration and bicarbonate hemodialysis.

**Methods:**

The transcriptomic profile was investigated in PBMCs isolated from eight patients on on-line hemodiafiltration and eight patients on bicarbonate hemodialysis by microarray analysis. The results were evaluated by statistical and functional pathway analysis and validated by real time PCR (qPCR) in an independent cohort of patients (on-line hemodiafiltration *N* = 20, bicarbonate hemodialysis *n* = 20).

**Results:**

Eight hundred and forty-seven genes were differentially expressed in patients treated with  on-line hemodiafiltration and bicarbonate hemodialysis. Thirty-seven functional gene networks were identified and atherosclerosis signaling was the top canonical pathway regulated by on-line hemodiafiltration. Among the genes of this pathway, on-line hemodiafiltration was associated with a reduced expression of Platelet-derived growth factor A chain (PDGF A), Clusterin, Monoamine Oxidase A, Interleukin-6 (IL-6) and Vascular Endothelial Growth Factor C (VEGF-)C and with an increase of Apolipoprotein E. qPCR confirmed the microarray results. Platelet derived growth factor AA (PDGF-AA), IL-6 and VEGF-C serum levels were significantly lower in the on-line hemodiafiltration group. Finally, 10 patients previously on bicarbonate hemodialysis  were switched to on-line hemodiafiltration and PBMCs were harvested after 6 months. The qPCR results from this perspective group confirmed the modulation of atherosclerotic genes observed in the cross-sectional analysis.

**Conclusions:**

Our data suggest that type of dialysis (on-line hemodiafiltration versus bicarbonate hemodialysis) may modulate the expression of several genes involved in the pathogenesis of atherosclerotic disease.

## Introduction

End-stage renal disease (ESRD) patients have a high cardiovascular mortality rate, which is substantially higher compared to the general population, particularly in young subjects [1, 2]. In this scenario, traditional risk factors do not entirely explain the observed burden of cardiovascular morbidity and mortality [3]. The presence of kidney dysfunction along with the renal replacement treatment with hemodialysis may affect the cardiovascular system in multiple ways, including accelerated progression of atherosclerosis, left ventricular hyperplasia and exacerbation of congestive heart failure [3]. ESRD patients on hemodialysis, in particular, develop fibrosis and calcification of the conduit arteries, leading to increased stiffness and accelerated atherosclerosis [4]. Among other ESRD-related risk factors, chronic inflammation might represent a key pathogenic modulator of the vascular disease observed in hemodialysis patients [5]. Uremia *per se* is a pro-inflammatory condition [6]. In addition, repeated contact between the blood and the hemodialysis membranes causes the activation of circulating immune cells and the subsequent expression and release of several pro-inflammatory and, potentially, atherogenic cytokines and growth factors [7]. Finally, retention of uremic toxins in patients with ESRD might negatively affect the cardiovascular system, also through the activation of the innate immune system [8]. These uremic toxins are poorly removed by hemodialysis techniques based on diffusion [9]. Conventional low and high-flux bicarbonate hemodialysis (BHD) provides diffusive clearance of low-molecular-weight solutes and has limited ability to remove middle-sized solutes [9]. In contrast, convective therapies, including hemofiltration and online-hemodiafiltration (OL-HDF) provide higher clearance of middle-size molecules [10].

We previously demonstrated that specific transcriptomic patterns of circulating immune cells characterize the systemic microinflammation occurring in uremic patients [11], whereas a specific immune transcriptomic profile discriminates dialyzed from un-dialyzed CKD patients [12]. In addition, we observed that transcriptomic inflammatory fingerprints are significantly different in hemodialysis and peritoneal dialysis. To date, no information is available concerning the potential influence of diffusive and convective hemodialysis treatments on the transcriptomic profile of circulating immune cells. Thus, the aim of our study was to identify, through a high-throughput approach, differences in gene expression profiles in peripheral blood mononuclear cells (PBMCs) of uremic patients on OL-HDF and BHD.

## Methods

### Patients

After obtaining written informed consent, we enrolled fifty-six hemodialysis patients into the present study based on the following inclusion criteria; stably on the same hemodialysis treatment (either OL-HDF or BHD) for at least 12 months, arterio-venous fistula (Qb > 300 ml/min) as vascular access, hemoglobin level ranging between 10 and 13 g/dL in the previous 3 months, absence of infections, neoplasia, auto-immune diseases, unstable heart failure, severe hypertension (systolic blood pressure > 180 or diastolic blood pressure > 110 mmHg), active liver diseases, severe malnutrition (albumin < 20 g/L), corticosteroid or immunosuppressive therapy. BHD subjects were treated with 240 min hemodialysis sessions three times per week using ultrapure bicarbonate-based dialysate and synthetic high-flux membrane. 1.8 m^2^ high-flux Helixone dialysers (FX80; Fresenius Medical Care, Bad Homburg, Germany) were used for conventional BHD and 1.8 m^2^ high-flux Helixone hemodiafilters (FX800; Fresenius Medical Care, Bad Homburg, Germany) were used for OL-HDF. OL-HDF was performed following the same BHD weekly schedule, using a blood flow rate of > 300 mL/min, a dialysate flow rate of 500 mL/min and synthetic high-flux dialysers. Ultrapure bicarbonate-buffered substitution volume produced on-line was infused in a post-dilution mode with a target convective volume of ≥ 22 L. The cohort included patients who were recruited over a period spanning three years from 2015 to 2018. Such progressive recruitment was carried out as consecutive patients met the inclusion criteria. We included patients matched for the clinical variables shown in Table 1. Among these patients, eight patients/treatment group were randomly selected and assigned to the microarray analysis group, the remaining forty patients represented the test group. After the initial analysis, 10 BHD patients from the test group were converted for clinical reasons (mainly hemodynamic instability) to OL-HDF. The shift to OL-HDF was spread out over the same period as required from a clinical standpoint. Molecular analyses were repeated in this group of patients six months after conversion.

**Table 1:** Main demographic and clinical features of the patients included in the study

	Microarray group		Test group		Switched group	
	BHD	OL-HDF	BHD	OL-HDF	BHD	OL-HDF
Patients (*n*)	8	8	20	20	10	10
Gender (M/F)	4/4	5/3	12/8	11/9	6/4	6/4
Age (years)	63.2 ± 4.0	60.7 ± 9	58.6 ± 11.4	59.1 ± 10.8	57.4 ± 10.4	–
Time on dialysis (months)	33.8 ± 12.4	31.7 ± 15.3	34.7 ± 11.2	32.2 ± 16.5	34.8 ± 10.2	–
BMI (kg/m2)	22.8 ± 0.7	21.8 ± 0.9	22.8 ± 0.2	23.7 ± 0.9	23.8 ± 0.1	–
Phosphorus (mg/dL)	4.9 ± 1.3	4.6 ± 1.1	4.8 ± 1.5	4.7 ± 0.8	4.6 ± 1.5	4.3 ± 0.8
Calcium (mg/dL)	9.02 ± 0.7	9.04 ± 0.6	8.9 ± 0.9	9.1 ± 0.4	9.0 ± 0.7	9.1 ± 0.3
PTH (ng/mL)	292.9 ± 110.3	280.9 ± 138.3	301.1 ± 157.3	275.9 ± 198.3	280.9 ± 160.3	265.1 ± 153.1
hsCRP (mg/dL)	0.9 ± 1.2	0.8 ± 1.5	0.8 ± 1.1	0.9 ± 1.0	0.8 ± 1.1	0.7 ± 1.0
Hemoglobin (g/dL)	11.5 ± 1.3	11.6 ± 1.7	11.3 ± 1.2	11.4 ± 1.6	11.4 ± 1.3	11.6 ± 1.4
Diabetes (*n*, %)	2 (25%)	3 (37.5%)	6 (30%)	7 (35%)	3 (30%)	3 (30%)
Hypertension (*n*, %)	6 (75%)	6 (75%)	17 (85%)	18 (90%)	8 (80%)	8 (80%)
Dyslipidemia (*n*, %)	4 (50%)	5 (62.5%)	11 (55%)	12 (60%)	6 (60%)	6 (60%)
Heart disease (*n*, %)	5 (62.5%)	5 (62.5%)	12 (60%)	11 (55%)	6 (60%)	6 (60%)
Cerebrovascular disease (*n*, %)	2 (25%)	2 (25%)	5 (25%)	4 (20%)	2 (20%)	2 (20%)
Peripheral vascular disease (*n*, %)	2 (25%)	2 (25%)	5 (25%)	4 (20%)	3 (30%)	3 (30%)

The study was approved by the institutional ethical board of the University Hospital “Policlinico Consorziale”, Bari, Italy (study number 4299) and it was performed according to the latest version of the declaration of Helsinki.

### PBMC isolation and RNA extraction

Twenty ml of whole blood were harvested from all patients at the time of enrollment, immediately before starting the dialysis session. PBMCs were isolated by density separation over a Ficoll-Paque™ gradient (GE Healthcare, Uppsala, Sweden). Total RNA was extracted automatically and qualitatively, and quantitatively analyzed through Agilent Bioanalyzer (Agilent Technologies, Santa Clara, CA). Only samples of good quality characterized by RNA integrity number (RIN) > 8 were used in the microarray experiment.

### Microarray experiment

For transcriptomic profiling, labeled cRNA was generated using the Low Input Quick Amp Labeling (LIQA) kit, according to the manufacturer’s protocols (Agilent Technologies), from RNA samples of 8 OL-HDF or 8 BHD. Gene expression data were obtained by the Agilent Feature Extraction software. Results of the microarray experiments are available in Gene Expression Omnibus (Series number: GSE129247). The differentially expressed genes were identified by applying a fold change ≥ 1.5 and *p* value < 0.05 after comparison of the two groups by *t* test (Moderate *t* test). Permutation analysis was applied to reduce the false discovery rate. Results were statistically analyzed using the software GeneSpring GX 12.5 in order to identify differentially expressed genes, and were functionally analyzed using the IPA software (www.ingenuity.com) that, based on the information in the literature, analyzes the molecular and biological functions in which the differentially expressed genes are included.

### Real time PCR

The results gathered by microarray analysis were validated by quantitative real time-PCR in an independent cohort of patients (20 OL-HDF or 20 BHD). Reverse transcription of total RNA (500 ng) was performed using the High Capacity cDNA Reverse Transcription Kit (Applied Biosystems, Foster City, CA), following the manufacturer’s instructions. The real time PCR experiments were performed in triplicate on the Light Cycler 96 Instrument (Roche, Mannheim, Germany) using the following PrimePCR SYBR Green Assay (BioRad): qHsaCED0038674 (GAPDH); qHsaCID0020314 (IL-6); qHsaCID0012475 (Clusterin); qHsaCID0023062 (Apolipoprotein E); qHsaCID0022048 (Mono-amino oxidase A); qHsaCID0005879 (PDGF A chain); qHsaCID0015147 (VEGF C). Relative quantification was obtained using a comparative Ct method as previously described [13].

### Elisa

Serum levels of 20 samples were collected from an independent cohort of patients (20 OL-HDF or 20 BHD). Serum levels of PDGF AA, IL-6 and VEGF C were measured using Biomarker Assay Service Labospace (LXSAHM-04 Human Magnetic Luminex Screening Assay).

### Statistical analysis

Microarray statistical analysis was performed as previously described [13–15]. Data are presented as mean ± standard deviation (SD) or median and interquartile range and compared by *t* test or Kruskall–Wallis test, as appropriate. A *p* < 0.05 was considered statistically significant.

## Results

The main demographic and clinical features of the subjects included in the study are summarized in Table 1.

**Fig. 1:**
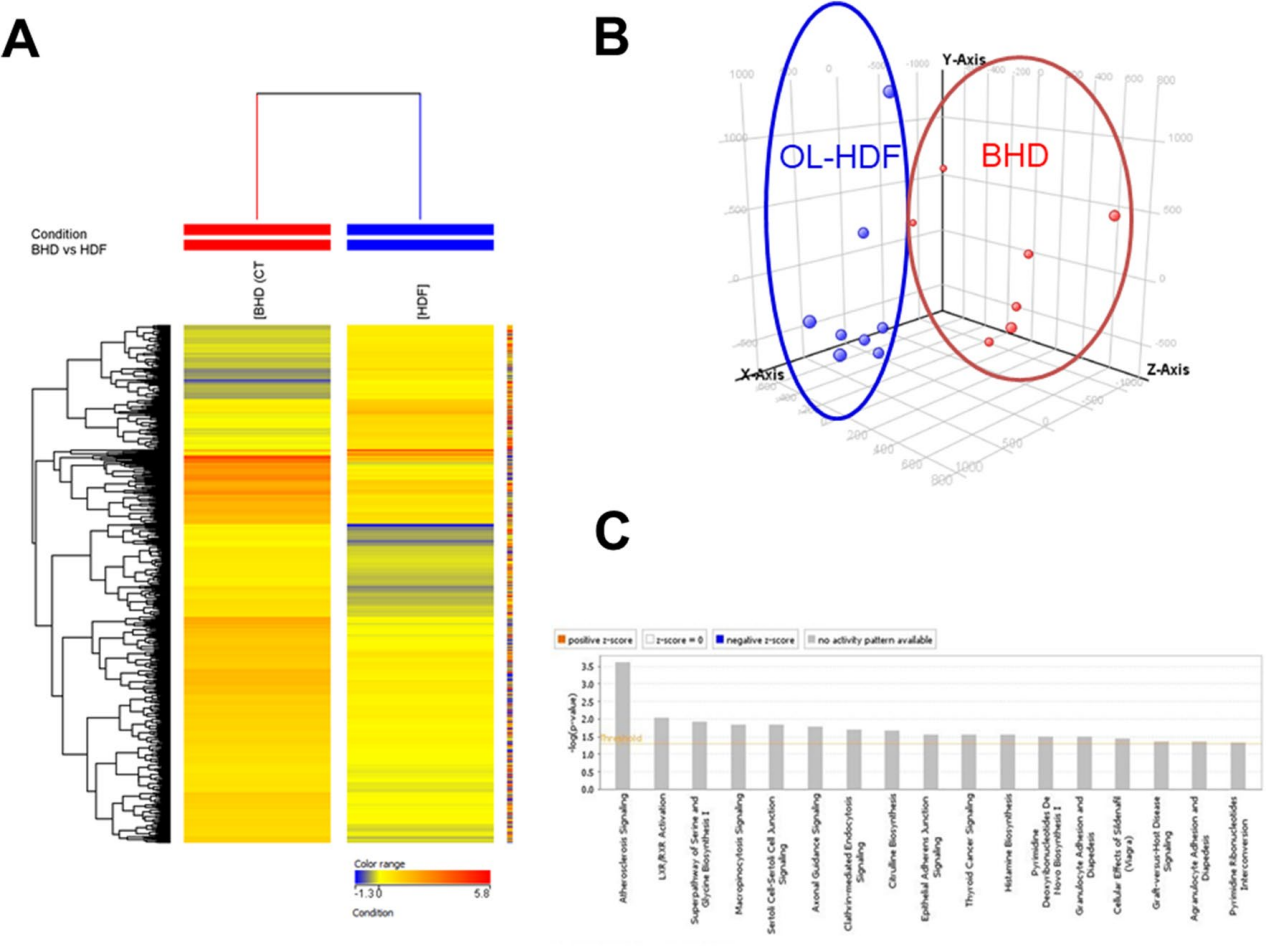
**a** Hierarchical clustering heat map of 868 differentially expressed probe sets. Patients are represented with a blue line at the top indicating OL-HDF (*n* = 8) or a red line indicating BHD (CTRL) (*n* = 8). The relative level of gene expression is depicted from lowest (blue) to highest (red). **b** Principal component analysis (PCA) of mRNAs discriminating OL-HDF and BHD subjects. PCA of the 868 differentially expressed genes clearly shows the degree of separation between the OL-HDF and BHD groups. OL-HDF (*n* = 8) and BHD (*n* = 8) patients are indicated in blue and red, respectively. **c** Top Canonical Pathways of differentially expressed genes in PBMCs of OL-HDF versus BHD patients. Histograms show the Canonical Pathways generated using IPA software, including all genes discriminating the two study groups

We compared the transcriptomic profiles of 8 ESRD patients treated by OL-HDF and eight patients undergoing standard BHD. According to an independent statistical algorithm and the estimated false discovery rate, we observed that 868 probe sets, corresponding to 847 genes, were differentially expressed in the comparison between OL-HDF and BHD. In 200 out of 847 genes the expression level was increased in OL-HDF (higher than 1.5 fold), whereas for 647 genes the mRNA abundance was reduced more than 1.5 fold in OL-HDF. Two dimensional hierarchical clustering and principal component analysis (PCA) demonstrate a highly significant separation of the two patient groups based on the 847 differentially expressed genes (Fig. 1a, b, respectively). Microarray data evaluation by ingenuity pathway analysis (IPA) identified 37 functional gene networks significantly associated with OL-HDF and revealed, among the most significant biological functions, an enrichment of transcripts related to cancer (*p* range = 6.82E-03-1.38E-13, 478 molecules); organismal injury and abnormalities (*p* range = 5.73E-03-5.63E-09, 255 molecules); inflammatory response (*p* range = 7.02E-03-3.27E-08, 119 molecules) and cardiovascular disease (*p* range = 7.09E-03-3.55E-06, 94 molecules). Finally and most interestingly, *Atherosclerosis signaling* was the top canonical pathway associated with BHD (Fig. 1c, *p* = 2.45 × 10^–4^).

**Fig. 2:**
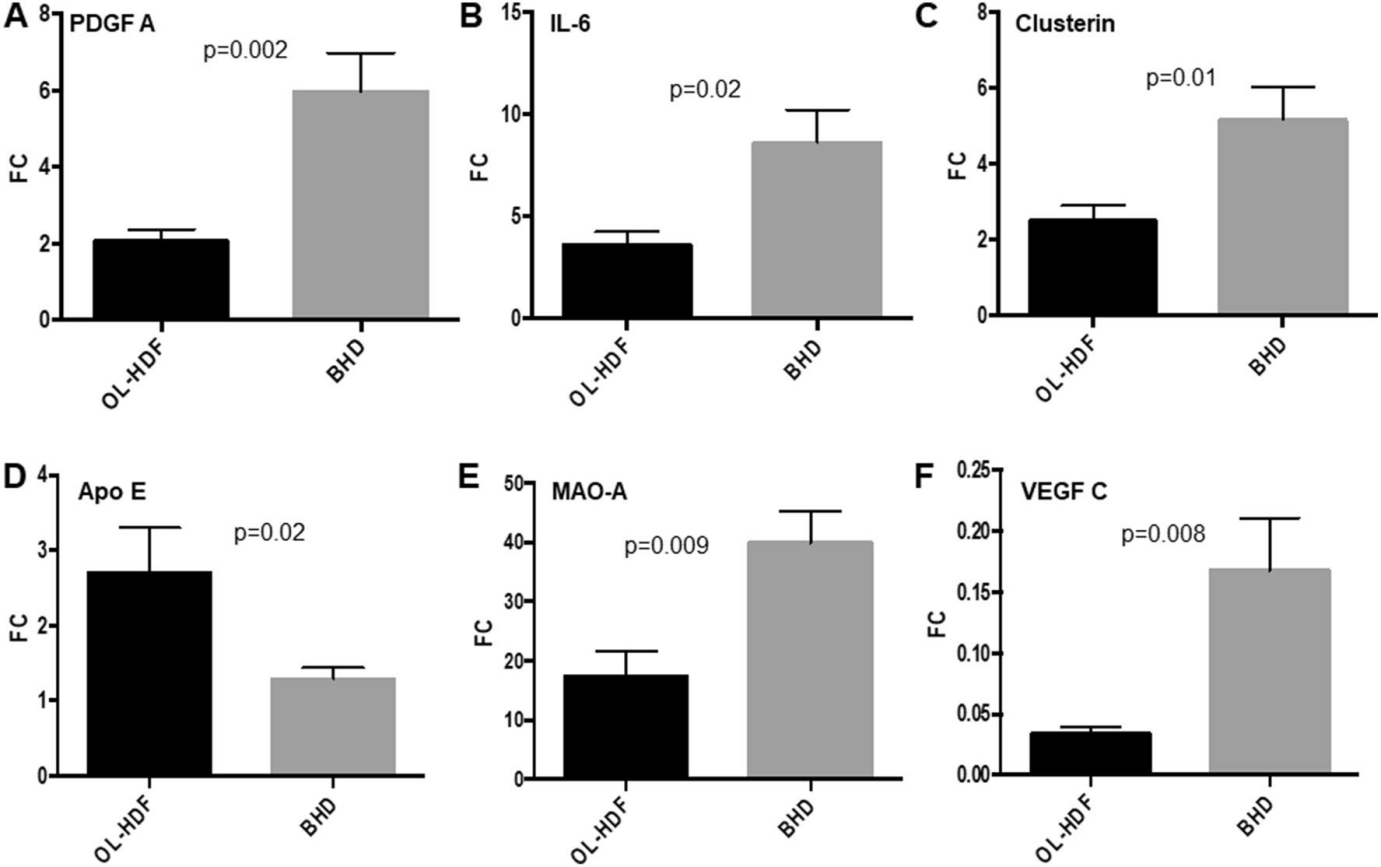
Validation by quantitative real-time PCR of the most highly deregulated genes identified by microarray experiments: PDGF AA chain (**a**), IL-6 (**b**), Clusterin (**c**), ApoE (**d**), MAO-A (**e**) and VEGF C (**f**). Expression levels were evaluated in PBMCs isolated from an independent group of OL-HDF (*n* = 20; black columns) and BHD patients (*n* = 20; gray columns). The histograms represent the mean ± SD of the expression of the selected gene. *FC* Fold change

**Fig. 3:**
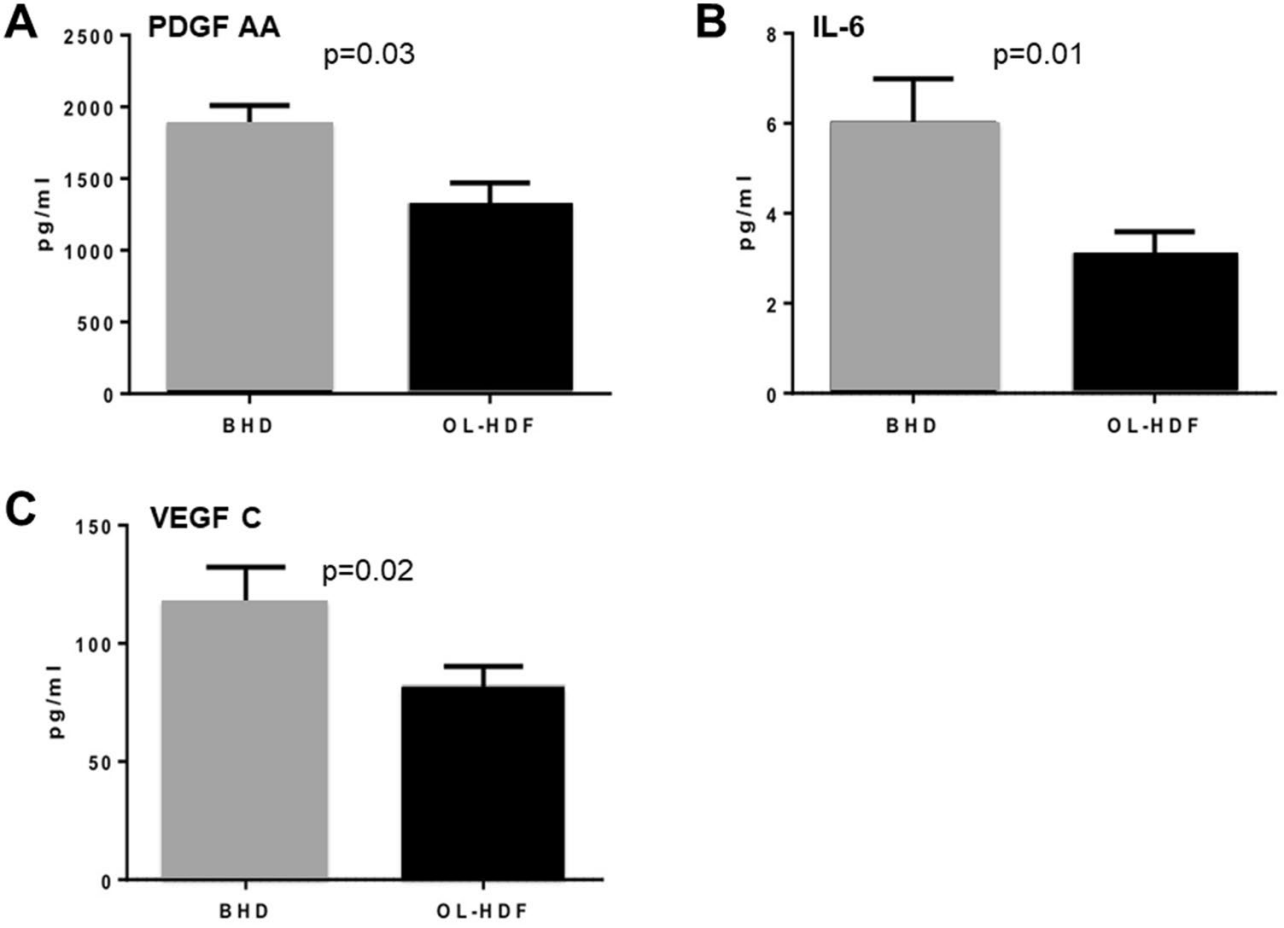
PDGF AA (**a**), IL-6 (**b**) and VEGF C (**c**) serum levels in an independent group of OL-HDF (*n* = 20*;* black columns) and BHD patients (*n* = 20; gray columns). Serum levels of the three cytokines were evaluated as described in the Methods section. The histograms represent the mean ± SD of the selected protein levels

**Fig. 4:**
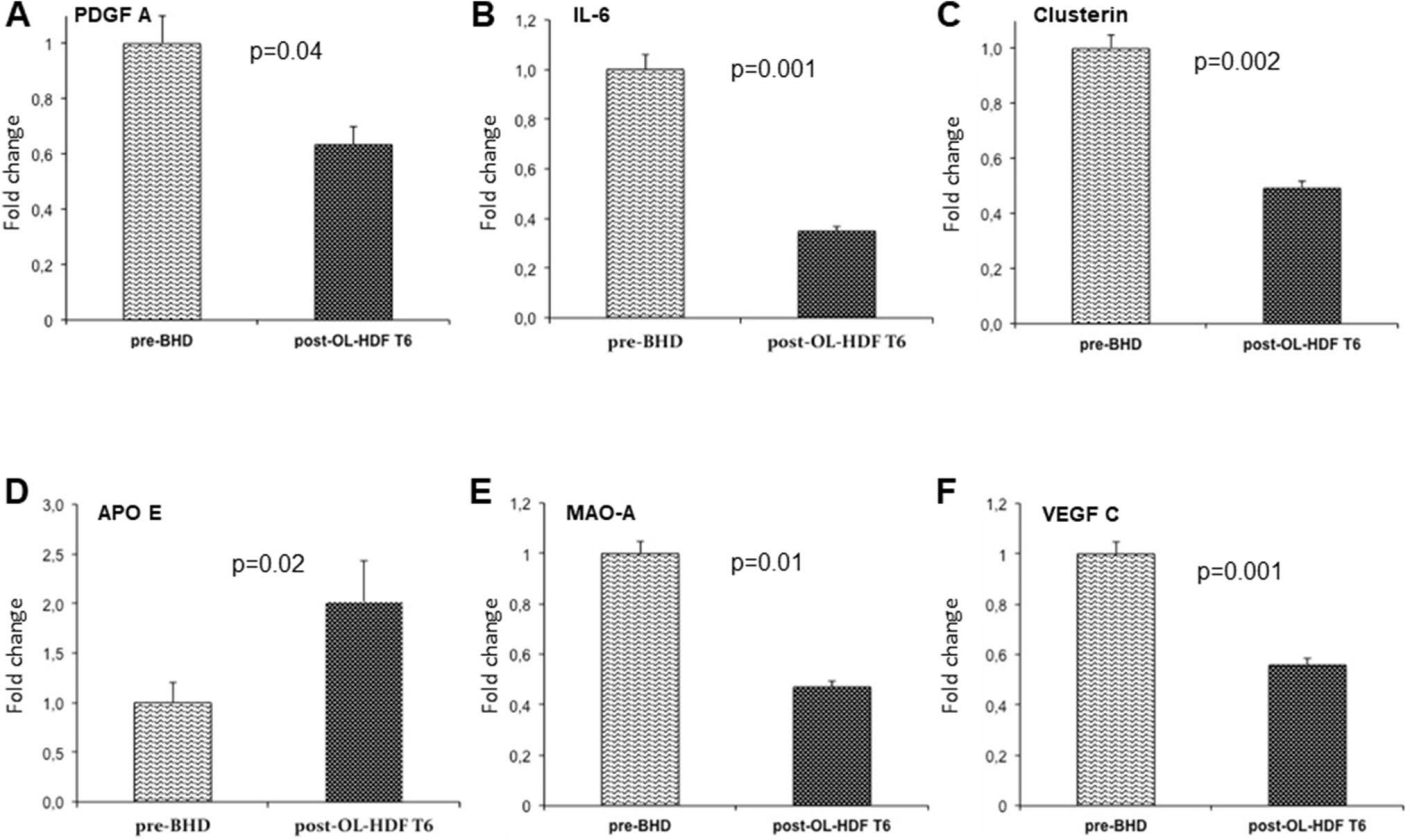
PDGF AA (**a**), IL-6 (**b**), Clusterin (**c**), ApoE (**d**), MAO-A (**e**) and VEGF C (**f**) gene expression in PBMC isolated from BHD patients (*n* = 10) before (gray columns) and six months after (black columns) the switch to OL-HDF. mRNA abundance was evaluated by qPCR. The histograms represent the mean ± SD of the expression of the selected gene

Since the consequences of accelerated atherosclerosis are the main cause of death in hemodialysis patients we focused on the main genes belonging to this pathway. In particular, we investigated the mRNA abundance, by quantitative real time PCR, of the following genes: *Platelet-derived growth factor A chain* (PDGF A chain, FC =  − 2.13), a well-known pathogenic mediator of atherosclerosis, *Interleukin-6* (IL-6, FC =  − 1.56), a pro-inflammatory cytokine which accelerates the progression of the atherosclerotic process, *Monoamine Oxidase A* (MAO-A, FC −2.43), a key source of oxidative stress, *Clusterin* (CLU, FC −2.14), a molecular chaperone involved in the regulation of oxidative stress, *Vascular Endothelial Growth Factor* (VEGF, FC −1.83) known to enhance the pathophysiologic mechanism of plaque formation and plaque destabilization, and *Apolipoprotein E* (APOE, FC =  + 1.69), which maintains overall plasma cholesterol homeostasis and modifies both macrophage- and T lymphocyte-mediated immune responses that contribute to atherosclerosis. Real time PCR of the six target genes in the test group confirmed the results of the microarray data (Fig. 2a-f). In addition, since some of the genes encode proteins that are released and may act on the vascular system in a paracrine fashion, we investigated the serum levels of PDGF AA, IL-6 and VEGF C in the same patients. As shown in Fig. 3a–c, the serum levels of the three cytokines were significantly lower in the patients treated by OL-HDF compared to BHD. Finally, to further demonstrate that the changes in the expression levels in the six target genes involved in atherogenesis were, indeed, due to the different hemodialysis treatment, 10 BHD patients that had been converted to OL-HDF were further evaluated 6 months after conversion. Evaluation in real time PCR of the six genes of interest confirmed their potential modulation by OL-HDF in this setting as well (Fig. 4 a-f).

## Discussion

In the current study, we identified for the first time, through a high-throughput approach, differences in PBMC gene expression profiles associated with OL-HDF and BHD and provided evidence suggesting that OL-HDF might modulate specific protein expression involved in atherosclerotic disease. It is conceivable that convective therapy, compared to conventional BHD, may also contribute to improving the endothelial and immune dysfunction frequently observed in patients undergoing hemodialysis treatment [16–18].

Archetypal cardiovascular risk factors have been associated with a high incidence of cardiovascular disease featuring HD patients [2, 3]. These traditional risk factors, however, appear to only partially explain the increased burden of cardiovascular risk in this population [1–3]. It is conceivable that the removal of middle molecules by high volume convection may modulate the activation of circulating immune cells, the primary players in the development and progression of atherosclerosis [8–10]. On this basis, using a transcriptomic approach we investigated the activation levels of circulating immune cells isolated from eight patients on OL-HDF and eight patients on BHD. We found PDGF A chain, IL-6, Monoamine Oxidase A, Clusterin, and VEGF C to be among the genes that were significantly down-regulated by OL-HDF.

PDGF and VEGF C are two growth factors with a well-established role in the pathogenesis of atherosclerosis. Ross et al. originally suggested PDGF as the main mediator of smooth muscle cell migration, activation and proliferation in response to endothelial cell stress, the first step in the development of atherosclerotic lesions [19]. A more recent view of the role of PDGF in the pathogenesis of atherosclerosis is linked to the increasing relevance in this setting of vessel wall inflammation. In this view, the main cells expressing and releasing PDGF are the infiltrating monocytes [20]. Inhibition of PDGF signaling significantly reduces the development of atherosclerotic lesions in APOE null mice, a well-established model of accelerated atherosclerosis [21]. Interestingly, we observed that OL-HDF in our patients causes a significant increase in APOE expression by circulating immune cells. This lipoprotein plays a key protective role in atherosclerosis by maintaining overall plasma cholesterol homeostasis and facilitating efficient hepatic uptake of lipoprotein remnants and directly modifies both macrophage- and T lymphocyte-mediated immune responses that contribute to atherosclerosis [22]. In addition, it has antioxidant, antiproliferative (smooth muscle cells, lymphocytes), anti-inflammatory, anti-platelet, and NO-generating properties [22].

On the other hand, VEGF C is a key growth factor for lymphatic endothelial cells and is significantly increased in human atherosclerotic lesions, where it is mainly expressed by infiltrating monocytes [23]. In addition, in APOE-deficient mice fed a high-fat-diet or normal chow for 16 weeks, levels of VEGF-A did not significantly differ between the two groups, whereas VEGF-C serum levels were significantly higher in high-fat-diet mice with advanced atherosclerosis and marked hypercholesterolemia [24]. In addition, Machnik et al. demonstrated that VEGF C expression and production is significantly increased by hypertonic skin accumulation in response to a salt load [25]. In this setting VEGF C plays a key role in reducing interstitial sodium retention, modulating endothelial nitric oxide synthesis and controlling blood pressure [25]. Interestingly, Sahutoglu et al. demonstrated that circulating VEGF C is significantly and directly associated with hypervolemia [26]. Thus, it is conceivable that the decreased levels of VEGF C in OL-HDF patients might be due, at least in part, to better volume control obtained with this technique.

VEGF C, PDGF A chain and APOE genes are strictly correlated in the pathogenesis of atherosclerosis, and their modulation by OL-HDF would suggest an anti-atherogenic effect of this treatment. In addition, since these three genes appear to act at different steps of the same pathogenic pathways it is conceivable that their contextual modulation may have a synergic effect on the development of vascular damage.

IL-6 is the key pro-inflammatory cytokine and its pathogenic role in the development of cardiovascular disease is well established. This cytokine not only plays a pivotal role in the pathogenesis and progression of atherosclerosis, but it has also been implicated in the remodeling observed after myocardial infarction [27, 28]. Interestingly, a recent meta-analysis including nine prospective studies with more than 9000 patients demonstrated that circulating IL-6 levels are independently associated with a greater risk of cardiovascular mortality in the general elderly population [29]. This observation was also confirmed in patients on hemodialysis in a further meta-analysis considering 22 studies [30]. Thus, the reduction observed in our patients on OL-HDF compared to BHD may further support the hypothesis of a protective effect of this treatment. Interestingly, our results confirm the observation by den Hoedt et al. that OL-HDF can significantly reduce systemic inflammation in hemodialysis patients [31].

The two other genes that are modulated by OL-HDF are less known in the pathogenesis of atherosclerosis although they might not be less important. Clusterin or apolipoprotein J is a molecular chaperone involved in several pathological conditions related to oxidative stress, including neurodegenerative diseases, cancers, inflammatory diseases and aging [32]. Although its pro or anti-atherogenic role has been largely discussed, there is compelling evidence that reduced expression, as observed in our patient population, might be protective. The main experimental support to this hypothesis is the observation derived from a study using double knock out mice for APOE and clusterin. In these mice the atherosclerotic lesions featuring APOE null animals were significantly reduced [33]. In addition, there is evidence that clusterin expression is significantly increased in human atherosclerotic lesions, suggesting a role for this protein in the development and progression of vascular injury [34]. On the other hand, MAO-A might represent an important source of oxidative stress, and its expression and/or activity might represent a major contributing factor to the development of pathologic left ventricular hypertrophy and heart failure [35]. Oxidative stress has been proposed as a non-traditional cardiovascular risk factor in uremia and has been well documented in hemodialysis patients [36].

The main limit of the present study is represented by the small sample size. However, confirmation of the observation also in a prospective cohort strengthens its  value. Considering the limited number of patients that were included and the short observation period of our study, we could not correlate the molecular changes with hard clinical outcomes. Thus, our observation should be considered more “phenomenological” than “mechanistic” and therefore it represents a proof-of-concept on which we aim to design a confirmatory cohort observational investigation with a larger patient population. On the other hand, the main strength of our report is the unbiased transcriptomic analysis. Using this methodological approach, our definition of a pathogenic hypothesis for the beneficial cardiovascular effect of OL-HDF does not suffer from any potential prejudice.

In conclusion, we demonstrate for the first time that OL-HDF may significantly reduce activation of the circulating immune cells modulating the expression of several pathogenic factors potentially involved in the development of atherosclerotic disease.

## References

[1] de Jager DJ, Grootendorst DC, Jager KJ (2009). Cardiovascular and non-cardiovascular mortality among patients starting dialysis. JAMA.

[2] Go AS, Chertow GM, Fan D, McCulloch CE, Hsu CY (2004). Chronic kidney disease and the risks of death, cardiovascular events, and hospitalization. N Engl J Med.

[3] Mathew RO, Bangalore S, Lavelle MP (2017). Diagnosis and management of atherosclerotic cardiovascular disease in chronic kidney disease: a review. Kidney Int.

[4] Shroff R, Long DA, Shanahan C (2013). Mechanistic insights into vascular calcification in CKD. J Am Soc Nephrol.

[5] Kooman JP, Dekker MJ, Usvyat LA (2017). Inflammation and premature aging in advanced chronic kidney disease. Am J Physiol Renal Physiol.

[6] Dai L, Golembiewska E, Lindholm B, Stenvinkel P (2017). End-stage renal disease, inflammation and cardiovascular outcomes. Contrib Nephrol.

[7] Gesualdo L, Pertosa G, Grandaliano G, Schena FP (1998). Cytokines and bioincompatibility. Nephrol Dial Transplant.

[8] Moradi H, Sica DA, Kalantar-Zadeh K (2013). Cardiovascular burden associated with uremic toxins in patients with chronic kidney disease. Am J Nephrol.

[9] Yu X (2017). The evolving patterns of uremia: unmet clinical needs in dialysis. Contrib Nephrol.

[10] Thomas G, Jaber BL (2009). Convective therapies for removal of middle molecular weight uremic toxins in end-stage renal disease: a review of the evidence. Semin Dial.

[11] Zaza G, Pontrelli P, Pertosa G (2008). Dialysis-related systemic microinflammation is associated with specific genomic patterns. Nephrol Dial Transplant.

[12] Zaza G, Granata S, Rascio F (2013). A specific immune transcriptomic profile discriminates chronic kidney disease patients in pre-dialysis from hemodialyzed patients. BMC Med Genomics.

[13] Rascio F, Pontrelli P, Accetturo M (2015). A type I interferon signature characterizes chronic antibody-mediated rejection in kidney transplantation. J Pathol.

[14] Dell'Oglio MP, Zaza G, Rossini M (2010). The anti-fibrotic effect of mycophenolic acid-induced neutral endopeptidase. J Am Soc Nephrol.

[15] Pontrelli P, Rascio F, Zaza G (2019). Interleukin-27 is a potential marker for the onset of post-transplant malignancies. Nephrol Dial Transplant.

[16] Jia P, Jin W, Teng J (2016). Acute effects of hemodiafiltration versus conventional hemodialysis on endothelial function and inflammation: a randomized crossover study. Medicine (Baltimore).

[17] Espi M, Koppe L, Fouque D, Thaunat O (2020). Chronic kidney disease-associated immune dysfunctions: impact of protein-bound uremic retention solutes on immune cells. Toxins.

[18] Kim HY, Yoo TH, Hwang Y (2017). Indoxyl sulfate (IS)-mediated immune dysfunction provokes endothelial damage in patients with end-stage renal disease (ESRD). Sci Rep.

[19] Ross R (1986). The pathogenesis of atherosclerosis - an update. N Engl J Med.

[20] Ross R, Masuda J, Raines EW (1990). Localization of PDGF-B protein in macrophages in all phases of atherogenesis. Science.

[21] Sano H, Sudo T, Yokode M (2001). Functional blockade of platelet-derived growth factor receptor-beta but not of receptor-alpha prevents vascular smooth muscle cell accumulation in fibrous cap lesions in apolipoprotein E-deficient mice. Circulation.

[22] Marais AD (2019). Apolipoprotein E in lipoprotein metabolism, health and cardiovascular disease. Pathology.

[23] Nakano T, Nakashima Y, Yonemitsu Y (2005). Angiogenesis and lymphangiogenesis and expression of lymphangiogenic factors in the atherosclerotic intima of human coronary arteries. Hum Pathol.

[24] Wada H, Ura S, Kitaoka S (2011). Distinct characteristics of circulating vascular endothelial growth factor-A and C levels in human subjects. PLoS ONE.

[25] Machnik A, Neuhofer W, Jantsch J (2009). Macrophages regulate salt-dependent volume and blood pressure by a vascular endothelial growth factor-C-dependent buffering mechanism. Nat Med.

[26] Sahutoglu T, Sakaci T, Hasbal NB (2017). Serum VEGF-C levels as a candidate biomarker of hypervolemia in chronic kidney disease. Medicine.

[27] Ridker PM (2016). From C-reactive protein to interleukin-6 to Interleukin-1: moving upstream to identify novel targets for atheroprotection. Circ Res.

[28] Huang M, Yang D, Xiang M, Wang J (2015). Role of interleukin-6 in regulation of immune responses to remodeling after myocardial infarction. Heart Fail Rev.

[29] Li H, Liu W, Xie J (2017). Circulating interleukin-6 levels and cardiovascular and all-cause mortality in the elderly population: a meta-analysis. Arch Gerontol Geriatr.

[30] Zhang W, He J, Zhang F (2013). Prognostic role of C-reactive protein and interleukin-6 in dialysis patients: a systematic review and meta-analysis. J Nephrol.

[31] den Hoedt CH, Bots ML, Grooteman MP (2014). Online hemodiafiltration reduces systemic inflammation compared to low-flux hemodialysis. Kidney Int.

[32] Jones SE, Jomary C (2002). Clusterin. Int J Biochem Cell Biol.

[33] Hamada N, Miyata M, Eto H (2011). Loss of clusterin limits atherosclerosis in apolipoprotein E-deficient mice via reduced expression of Egr-1 and TNF-α. J Atheroscler Thromb.

[34] Mackness B, Hunt R, Durrington PN, Mackness MI (1997). Increased immuno-localization of paraoxonase, clusterin, and apolipoprotein A-I in the human artery wall with the 35 progression of atherosclerosis. Arterioscler Thromb Vasc Biol.

[35] Santin Y, Sicard P, Vigneron F (2016). Oxidative stress by monoamine oxidase-a impairs transcription factor EB Activation and autophagosome clearance, leading to cardiomyocyte necrosis and heart failure. Antioxid Redox Signal.

[36] Cariello M, Simone S, Loverre A (2012). Coagulation activation is associated with nicotinamide adenine dinucleotide phosphate oxidase-dependent reactive oxygen species generation in hemodialysis patients. Antioxid Redox Signal.

